# A novel DNA methylation panel accurately detects colorectal cancer independently of molecular pathway

**DOI:** 10.1186/s12967-018-1415-9

**Published:** 2018-02-27

**Authors:** Micaela Freitas, Fábio Ferreira, Sónia Carvalho, Fernanda Silva, Paula Lopes, Luís Antunes, Sofia Salta, Francisca Diniz, Lúcio Lara Santos, José Flávio Videira, Rui Henrique, Carmen Jerónimo

**Affiliations:** 1 Cancer Biology & Epigenetics Group-Research Center (CI-IPOP), Research Center-LAB 3, Portuguese Oncology Institute of Porto (IPO Porto), F Bdg, 1st Floor, Rua Dr António Bernardino de Almeida, 4200-072 Porto, Portugal; 2 Departments of Pathology, Portuguese Oncology Institute of Porto (IPO Porto), Porto, Portugal; 3 Departments of Epidemiology, Portuguese Oncology Institute of Porto (IPO Porto), Porto, Portugal; 4 Departments of Surgical Oncology, Portuguese Oncology Institute of Porto (IPO Porto), Porto, Portugal; 5 Department of Pathology and Molecular Immunology, Institute of Biomedical Sciences Abel Salazar (ICBAS), University of Porto, Rua de Jorge Viterbo Ferreira n.º 228, 4050-313 Porto, Portugal

**Keywords:** Colorectal cancer, Gene promoter methylation, Biomarkers, Diagnosis, Prognosis

## Abstract

**Background:**

Colorectal cancer (CRC) is one of the most incident cancers, associated with significant morbidity and mortality, and usually classified into three main molecular pathways: chromosomal instability, microsatellite instability (MSI) and CpG island methylator phenotype (CIMP). Currently, available screening methods are either costly or of limited specificity, impairing global implementation. More cost-effective strategies, including DNA methylation-based tests, might prove advantageous. Although some are already available, its performance is suboptimal, entailing the need for better candidate biomarkers. Herein, we tested whether combined use of *APC*, *IGF2*, *MGMT*, *RASSF1A*, and *SEPT9* promoter methylation might accurately detect CRC irrespective of molecular subtype.

**Methods:**

Selected genes were validated using formalin-fixed paraffin-embedded tissues from 214 CRC and 50 non-malignant colorectal mucosae (CRN). Promoter methylation levels were assessed using real-time quantitative methylation-specific PCR. MSI and CIMP status were determined. Molecular data were correlated with standard clinicopathological features. Diagnostic and prognostic performances were evaluated by receiver operator characteristics curve and survival analyses, respectively.

**Results:**

Except for IGF2, promoter methylation levels were significantly higher in CRC compared to CRN. A three-gene panel (*MGMT*, *RASSF1A*, *SEPT9*) identified malignancy with 96.6% sensitivity, 74.0% specificity and 91.5 positive predictive value (area under the curve: 0.97), independently of tumor location, stage, and molecular pathway.

**Conclusions:**

Combined promoter methylation analysis of *MGMT*/*RASSF1A/SEPT9* displays a better performance than currently available epigenetic-based biomarkers for CRC, providing the basis for the development of a non-invasive assay to detect CRC irrespective of the molecular pathway.

**Electronic supplementary material:**

The online version of this article (10.1186/s12967-018-1415-9) contains supplementary material, which is available to authorized users.

## Background

Colorectal cancer (CRC) is the third most incident and the fourth leading cause of cancer-related death by cancer, worldwide [1]. The primary molecular pathways involved in CRC carcinogenesis are chromosomal instability (CIN), microsatellite instability (MSI) and CpG island methylator phenotype (CIMP), accounting for nearly 85, 15 and 10–40% of all sporadic cases, respectively [2, 3]. CIN and MSI represent two levels of genetic instability, a subtle one affecting only DNA sequences, MSI-high (MSI-H), and a gross one, affecting portions or entire chromosomes, i.e., CIN. These forms of instability are considered mutually exclusive: a CRC with CIN is most likely microsatellite stable (MSS) [4, 5]. In sporadic CRC, the most common cause of DNA mismatch repair (MMR) defects, leading to MSI-H, is aberrant bi-allelic *MLH1* aberrant promoter methylation. *MLH1* is also one of the markers often used to define CIMP, and, thus, an overlap between CIMP and MSI-H exists [2]. Two different panels have frequently been used to define CIMP, the classic panel proposed by An et al. (*MLH1*, *CDKN2A* (*p16*), *MINT1, MINT*2, and *MINT31*) and the panel designed by Weisenberger et al. (*CACNA1G*, *IGF2*, *NEUROG1*, *RUNX3*, and *SOCS1*) [6, 7]. Another categorization subdivides tumors into CIMP-High (CIMP-H), CIMP-Low (CIMP-L) and CIMP-negative (CIMP-0), each of them associated with different features [8]. Thus, different panels and/or a number of markers have been tested, and no consensus has been reached.

Several non-CIMP related methylation DNA targets have been found to distinguish malignant from non-malignant colorectal tissues [9]. Indeed, several studies have been conducted to identify a methylation biomarker or a panel of biomarkers with high sensitivity and specificity to be used in diagnosis and prognostication of CRC, but none has been validated [7, 10–21]. Moreover, only a few DNA methylation biomarkers intended for CRC detection are commercially available, including *ColoVantage*^®^, *EpiproColon*^®^ 2.0 and Abbott RealTime mS9, which are blood-based tests based on *septin 9* (*SEPT9*) promoter methylation [12, 18]. Nonetheless, the value of *SEPT9* promoter methylation as a biomarker has been questioned by several authors [22]. Because currently available screening methods are either costly or of limited specificity, impairing global implementation, DNA methylation-based tests are likely to be more cost-effective. Owing to the suboptimal performance of commercially available epigenetic tests, validation of better candidate biomarkers, which may detect CRC irrespective of molecular subtype, is warranted.

Based on an exhaustive literature review to select potentially useful gene promoters with the ability to discriminate malignant from non-malignant colorectal tissues, enabling its future testing in blood samples, we selected four genes hypermethylated in CRC [Adenomatous polyposis coli (*APC*) [13, 14], *O*-6-methylguanine-DNA methyltransferase (*MGMT*) [15, 23], Ras association domain family 1—isoform A (*RASSF1A*) [16] and Septin 9 (*SEPT9*) [12, 17, 18] ] and one gene hypomethylated in CRC [Insulin-like growth factor 2 *(IGF2*) [19–21]], for validation in a large cohort of CRCs, in which CIMP and MSI status were also determined. Furthermore, the potential prognostic value of gene promoter methylation was also assessed.

## Methods

### Patients and samples

A total of 214 CRC (110 colonic and 104 rectal cancers) from patients consecutively diagnosed and treated with surgical resection between 2000 and 2012 at Portuguese Oncology Institute of Porto (IPO Porto), Portugal, were included in this study (Table 1). Fifty samples of non-cancerous colorectal mucosa (CRN) from individuals with no evidence of CRC or other gastrointestinal tract cancer were used as controls (Additional file 1: Table S1). All samples corresponded to formalin-fixed paraffin-embedded (FFPE) tissues archived at the Department of Pathology of IPO Porto. Haematoxylin and eosin (H&E) stained tissue sections were reviewed and classified by an experienced pathologist according to current WHO classification (2010). Representative tumor areas were delimitated for further microdissection. Relevant clinical data were collected from medical charts, and tumor staging was performed using the American Joint Committee on Cancer (AJCC) criteria. This study was approved by the institutional ethics committee (CES 120/015).

**Table 1:** Clinicopathologic features of CRC patients by tumor location

Characteristic	Total (n = 214)	Colon (n = 110)	Rectum (n = 104)
Age (years) mean (range)	60.35 (25–80)	60.82 (25–80)	59.80 (31–80)
Gender			
Female	74 (34.6%)	42 (38.2%)	32 (30.8%)
Male	140 (65.4%)	68 (61.8%)	72 (69.2%)
Stage			
I/II	52 (24.3%)	24 (21.8%)	28 (26.9%)
III	52 (24.3%)	20 (18.2%)	32 (30.8%)
IV	108 (50.5%)	64 (58.2%)	44 (42.3%)
Unknown	2 (0.9%)	2 (1.8%)	–
Tumor differentiation			
Well	4 (1.9%)	1 (0.9%)	3 (2.9%)
Moderate	123 (57.5%)	79 (71.8%)	44 (42.3%)
Poor	5 (2.3%)	4 (3.6%)	1 (1.0%)
Not assessable	82 (38.3%)	26 (23.6%)	56 (53.8%)
*KRAS* mutation status			
Wildtype	116 (54.2%)	60 (54.5%)	56 (53.9%)
Mutated	84 (39.3%)	46 (41.8%)	38 (36.5%)
Not available	14 (6.5%)	4 (3.6%)	10 (9.6%)
MSI			
MSI-H	8 (3.7%)	8 (7.3%)	0 (0%)
MSI-L/MSS	206 (96.3%)	102 (92.7%)	104 (100%)
CIMP			
CIMP-positive	18 (8.5%)	6 (5.6%)	12 (11.5%)
CIMP-negative	193 (91.5%)	101 (94.4%)	92 (88.5%)
Neoadjuvant treatment			
Yes	69 (32.2%)	16 (14.5%)	53 (51.0%)
No	145 (67.8%)	94 (85.5%)	51 (49.0%)
Adjuvant treatment			
Yes	171 (80.0%)	89 (80.9%)	82 (78.8%)
No	45 (20.0%)	21 (19.1%)	22 (21.2%)

*CIMP* CpG island methylator phenotype, *MSI* microsatellite instability, *MSI-H* MSI high, *MSI-L* MSI low, *MSS* microsatellite stable

### Immunohistochemistry

Assessment of MSI status was accomplished through an immunohistochemical assessment of MLH1, MSH2, MSH6, and PMS2 expression, performed as previously described [24].

### Quantitative DNA methylation analysis (qMSP)

DNA was extracted from FFPE sections that contained at least 70% neoplastic cells, using phenol–chloroform conventional method as described previously Ramalho-Carvalho et al. [25]. DNA was quantified using NanoDrop ND-1000^®^ (NanoDrop Technologies, DE, USA) spectrophotometer and modified with sodium bisulfite, using the EZ DNA Methylation-Gold™ Kit (Zymo Research, Orange, CA, USA) according to manufacturer’s instructions. Bisulphite-treated DNA was used as a template for qMSP using specific primers for the target genes [*APC*, *IGF2*, *MGMT*, *RASSF1A* and *SEPT9* (Additional file 1: Table S2)] and CIMP markers (*CDKN2A*, *MLH1 MINT1*, *MINT2* and *MINT31* [7, 26]). Fluorescence-based real-time PCR assays were performed in 384-well plates in a LightCycler 480 II (Roche, Germany) using KAPA SYBR FAST qPCR Master Mix (Kapa Biosystems, USA). All the samples were run in triplicate and melting curve analysis was performed. Serial dilutions of modified CpGenome™ Universal Methylated DNA was used to generate a standard curve, and relative methylation levels were calculated as the ratio between the target gene mean quantity and *β*-*actin,* the reference gene, mean quantity, multiplied by 1000 for easier tabulation.

For CIMP status evaluation, each of the five markers composing the classical CIMP panel analyzed was considered methylated if the value of the previously described ratio was higher than any of the ratio values for the selected control samples and more significant than 25th percentile. Tumors were considered CIMP+ when more than one gene promoter was found hypermethylated.

### Statistical analysis

Methylation levels and clinical features were compared within groups using non-parametric tests (Mann–Whitney U test or Kruskall–Wallis test, as appropriate). Clinicopathological variables were compared to CIMP *status* using Chi square test or Fisher’s exact test, as applicable. Diagnostic performance of promoter methylated genes was not assessed in patients who underwent neoadjuvant treatments. ROC curves were constructed for each gene, and the best gene combination was assessed. For each panel were computed the specificity, sensitivity and accuracy as well as positive predictive value (PPV) and negative predictive value (NPV) were computed for each panel. The panel was considered positive for a specific sample when at least one of the genes was positive in the individual model.

Survival analysis was performed for disease-free survival (DFS) and disease-specific survival (DSS) through Kaplan–Meier method, and the prognostic significance of clinicopathological variables (age, gender, stage, tumor location, histology, KRAS mutation, MSI, and CIMP status) and methylation biomarkers were assessed using the two-sided Log-rank test to compare survival curves. Methylation levels were categorized using the 25th percentile for *IGF2* and the 75th percentile for the other genes. The reference groups considered were non-hypomethylated and non-hypermethylated, respectively. Multivariable analysis was carried out using a Cox proportional hazard model. Statistical analysis was performed using SPSS Statistics 22 (IBM, USA), and graphics were assembled using GraphPad Prism 6 (GraphPad Software, USA). A *P* value < 0.05 was considered statistically significant.

## Results

### Characteristics of patient population

Detailed characterization of the patient population is depicted in Table 1. Among the 214 CRC patients enrolled in this study, most were male (65.4%), and the median age was 60.35 years. Most of the tumors were in the colon (51.4%) and were at stage IV (50.5%). Neoadjuvant treatment was performed by 32.2% of patients, with a predominance of those with rectal location (51.0% vs. 14.5% for colonic location). Nevertheless, a similar proportion of patients with colonic vs. rectal cancer received adjuvant therapy (19.1 and 21.2%, respectively). Overall, *KRAS* mutations were identified in 39.3% of patients.

### MSI and CIMP status

Concerning MSI status, assessed by immunohistochemistry, only eight cases were considered MSI-H (3.7%), all localized in the colon. Regarding CIMP markers, *MINT31* showed the highest methylation frequency, whereas *MLH1* displayed the lowest, with 15.2 and 0.9%, respectively. Methylation frequencies of the remaining genes were 11.4% for *CDKN2A*, 14.7% for *MINT2* and 6.6% for *MINT1*. When methylation of all genes/loci were grouped based on methylation of 0 or one marker versus > 1 marker for the CIMP phenotype, 18 patients were classified as CIMP-positive (8.5%), and 193 patients were defined as CIMP-negative (91.5%).

### Gene promoter methylation levels and clinicopathological correlates

**Fig. 1:**
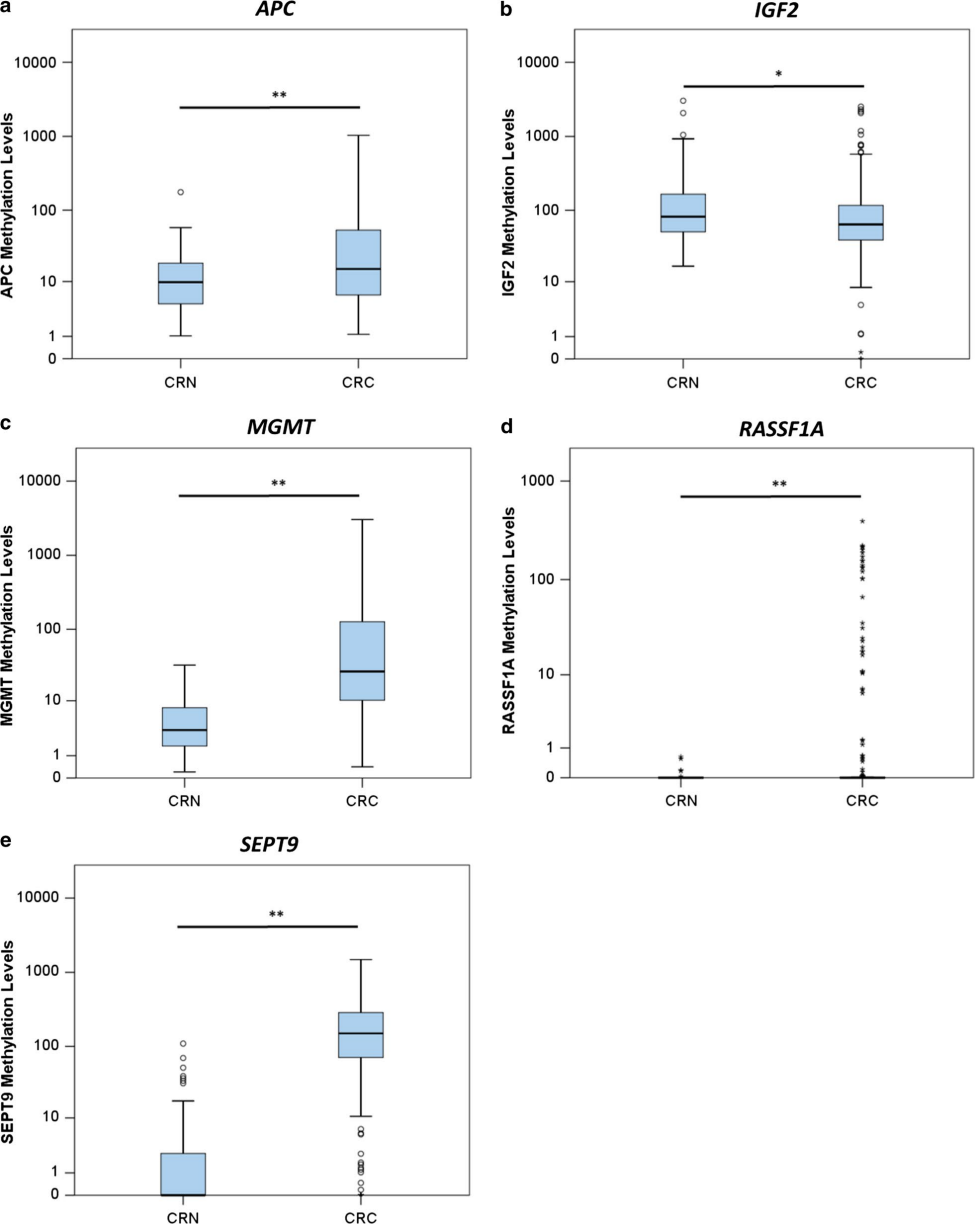
Distribution of *APC* (**a**), *IGF2* (**b**), *MGMT* (**c**), *RASSF1A* (**d**) and *SEPT9* (**e**), promoter methylation levels in normal (CRN) and neoplastic tissue (CRC). (Mann–Whitney U Test, *P < 0.05; **P < 0.01)

*APC, MGMT, SEPT9* and *RASSF1A* methylation levels were significantly higher in CRC compared to normal tissues (*P* = 0.005, *P* < 0.001, *P* < 0.001 and *P* = 0.002, respectively), whereas *IGF2* methylation levels were significantly lower in CRC (*P* = 0.025), as expected (Fig. 1). Although no significant association was found between any of the five promoter methylation levels and patients’ age, *MGMT* methylation levels were significantly higher in female patients compared to males both in the colon (*P* = 0.048) and rectum (*P* = 0.049).

Concerning tumour location, *MGMT*, *SEPT9,* and *RASSF1A* methylation levels were significantly higher in colon cancer (proximal and distal) patients (*P* = 0.000, *P* = 0.000 and *P* = 0.002, respectively), compared to CRN, whereas *APC*, *MGMT* and *SEPT9* displayed significantly higher promoter methylation levels in rectal cancer (*P* = 0.018, *P* = 0.0003 and *P* = 0.002, respectively). Moreover, higher *SEPT9* methylation levels were found in colon cancer than in rectal cancer patients (*P* = 0.021). Interestingly, for rectal cancer, *SEPT9* methylation levels were significantly higher in stage IV than in stages I, II or III (*P* = 0.001), whereas both *MGMT* and *SEPT9* methylation levels were significantly lower in patients that underwent neoadjuvant treatment (*P* = 0.012 and *P* = 0.002, respectively).

Moreover, and except for *APC* promoter methylation levels that were significantly higher in MSI-H tumors (*P* = 0.012), no additional significant differences were found for the remaining genes. Furthermore, no associations were found between gene promoter methylation levels and CIMP *status*.

### Diagnostic performance

**Fig. 2:**
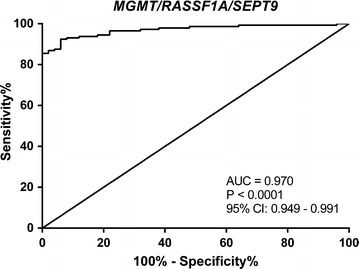
Receiver operating characteristic curve analysis considering patients without neoadjuvant treatment (N = 145) for *MGMT/RASSF1A/SEPT9*panel methylation with an AUC of 0.970. AUC, area under curve

Overall, the best performance was depicted by *SEPT9* followed by *MGMT* (AUCs of 0.950 and 0.894, respectively). Indeed, *SEPT9* promoter hypermethylation levels identified CRC with 85.5% sensitivity, 94.0% specificity, 97.6% PPV and 69.1% NPV. Considering the several combinations of genes in a panel, the best performance was accomplished by *MGMT/RASSF1A/SEPT9*, with AUC of 0.970, 96.6% sensitivity, 74.0% specificity, 91.5% PPV and 88.1% NPV (Fig. 2 and Table 2).

**Table 2:** Performance of epigenetic biomarkers for CRC’s detection in tissue samples

Gene/panels	Sensitivity (n/N)	Specificity (n/N)	AUC	PPV	NPV	Accuracy
*SEPT9*	85.5 (124/145)	94.0 (3/50)	0.950	97.6	69.1	87.7
*MGMT/SEPT9*	93.8 (136/145)	82.0 (9/50)	0.964	93.8	82.0	90.8
*MGMT/RASSF1A/SEPT9*	96.6 (140/145)	74.0 (13/50)	0.970	91.5	88.1	90.8

Sensitivity, Specificity, PPV and NPV values are represented in percentage

*AUC* Area under the curve, *NPV* negative predictive value, *PPV* positive predictive value

Importantly, the *MGMT/RASSF1A*/*SEPT9* panel detect cancer both in the colon (proximal and distal) and rectum with a sensitivity of 95.7% for colon and 98.0% for rectum. Furthermore, the panel was also able to identify tumors at any disease stage with similar efficiency (100, 94.2, 95.9% for stage I/II, stage III and stage IV, respectively), regardless of CIMP and MSI status, further supporting its value for CRC detection (Table 3).

**Table 3:** *SEPT9* promoter methylation and 3-gene panel sensitivity values for CRC detection according with location, stage and CIMP and MSI *status*

Diagnosis group	*SEPT9*	*SEPT9*, *MGMT*, *RASSF1A*
Location		
Colon	84.0 (79/94)	95.7 (90/94)
Proximal	86.7 (26/30)	93.3 (28/30)
Distal	82.8 (53/64)	96.9 (62/64)
Rectum	88.2 (45/51)	98.0 (50/51)
Stage		
Stage I and II	91.4 (32/35)	100 (35/35)
Stage III	80.0 (28/35)	94.2 (33/35)
Stage IV	84.9 (62/73)	95.9 (70/73)
CIMP		
CIMP-negative	85.5 (112/131)	96.9 (127/131)
CIMP-positive	90.9 (10/11)	100 (11/11)
MSI		
MSI-L/MSS	85.4 (117/137)	97.1 (133/137)
MSI-H	87.5 (7/8)	87.5 (7/8)

*CIMP* CpG island methylator phenotype, *MSI* microsatellite instability, *MSI-H* MSI high, *MSI-L* MSI low, *MSS* microsatellite stable, *n* number of positive cases, *N* total of cases in each group

### Survival analysis

Considering all CRC cases, *SEPT9* promoter methylation levels independently predicted for better DSS [Hazard Ratio (HR) = 0.673, 95% Confidence Interval (CI) 0.469–0.965] while age 60 years or higher and stage IV independently predicted for worse DSS (HR = 1.476, 95% CI 1.085–2.008 and HR = 1.862, 95% CI 1.295–2.677, respectively). Specifically, in colon cancer, *SEPT9* hypermethylation was significantly associated with better prognosis (HR = 0.472, 95% CI 0.276–0.806 and HR = 0.447, 95% CI 0.269–0.744, respectively), whereas age 60 years or higher was associated with worse DSS (HR = 1.730, 95% CI 1.079–2.773). Moreover, proximal colon tumors displayed worse prognosis (HR = 1.879, 95% CI 1.174–3.007). On the opposite, in rectal cancer, no associations were found between methylation levels or standard clinicopathological parameters and prognosis (Table 4).

**Table 4:** Multivariable Cox regression analysis of disease specific survival

Variable	Hazards ratio (95% CI)	*P* value
Colon and rectal samples (N = 214)		
Age (< 60 vs. ≥ 60)	1.476 (1.085–2.008)	*0.013*
Stage (I/II vs. III)	1.007 (0.657–1.544)	0.974
Stage (I/II vs. IV)	1.862 (1.295–2.677)	*0.001*
Neoadjuvant treatment (no vs. yes)	1.212 (0.879–1.673)	0.241
* SEPT9* methylation (non-Hyper. vs. hypermethylated)	0.673 (0.469–0.965)	*0.031*
Colon samples (N = 110)		
Age (< 60 vs. >=60)	1.730 (1.079–2.773)	*0.023*
Stage (I/II vs. III)	0.966 (0.486–1.922)	0.922
Stage (I/II vs. IV)	1.454 (0.859–2.463)	0.164
Neoadjuvant treatment (no vs. yes)	1.237 (0.677–2.259)	0.490
Tumor location (distal vs. proximal)	1.879 (1.174–3.007)	*0.009*
SEPT9 methylation (non-Hyper. vs. hypermethylated)	0.472 (0.276–0.806)	*0.006*

*95% CI* 95% confidence interval, *CRC* colorectal cancer, *non-Hyper.* non-hypermethylated

Italics values indicate statistically significant (*P* < 0.05)

## Discussion

Colorectal cancer is one of the most common and lethal malignancies, especially in developed countries [1]. Currently, CRC screening options mostly rely on fecal occult blood testing and endoscopy. Nevertheless, these strategies meet with significant limitations (e.g., low accuracy and higher cost, respectively) which impair its broader implementation [27]. Some DNA methylation-based biomarkers were developed and have been approved by FDA (Food and Drug Administration), including ColoVantage^®^, Epi proColon^®^, and ColoSure^®^, but are still not fully implemented in clinical practice. Whereas the former two are based on *SEPT9* methylation [12, 18], the latter is based on *Vimentin* methylation [28]. However, *SEPT9* promoter methylation performance in CRC detection has been recently questioned [22]. As for *Vimentin* promoter methylation, this test is used in combination with colonoscopy, but sensitivity is rather variable, ranging from 38 to 88% [28, 29]. Thus, alternative screening test is demanded to increase population adherence and perfect detection accuracy.

Globally, the distribution of promoter methylation levels for the five gene promoters tested among CRC and CRN tissues paralleled those of previous reports. Indeed, *IGF2* methylation levels were significantly lower in CRC than in CRN, in accordance a previous study [19], whereas the remainder genes showed significantly higher methylation levels in CRC, as reported by others [14, 15, 17, 30, 31]. Interestingly, *SEPT9* and *MGMT* methylation levels were significantly lower in rectal cancer patients that underwent neoadjuvant treatment, which is in line with studies in which ionizing radiation exposure induced global hypomethylation [32, 33], including for colon cancer [34].

Regarding the use of gene promoter methylation as CRC biomarker, the best detection performance was disclosed by *SEPT9* promoter methylation, followed by *MGMT*. Remarkably, *SEPT9* validity estimates were similar to those of the trademark assays using *SEPT9* methylation [12, 35], further confirming its biomarker potential. Nonetheless, *MGMT* displayed better performance (77.2% sensitivity, 84.0% specificity) than previously reported (46–53% sensitivity, 74–100% specificity), especially concerning sensitivity [23, 36]. This might be due not only to differences in the population under study, but also differences in the methodological approaches. The same might apply to *RASSF1A* performance, which disclosed lower sensitivity (33.1% vs. 81.0%) but higher specificity (90.0% vs. 51.0%) [16], although the definition of the cutoff value might also significantly impact on performance. Nonetheless, a combination of these three gene promoters significantly improved diagnostic performance when compared with previously published panels (Table 5). In our experience, the use of gene promoter panels, usually comprising two to four genes, improves sensitivity, without compromising specificity, as previously demonstrated for prostate and breast cancers, especially when tested in liquid biopsies [37, 38]. Thus, the use of this gene panel might augment the performance of approved DNA methylation-based assays.

**Table 5:** Comparison of sensitivity and specificity of previously published panels with values obtained for this new panel

Panels	Sensitivity (%)	Specificity (%)	AUC	Accuracy	Specimen type	Methods	References
*NGFR*; *SEPT9*; *TMEFF2*	NA	NA	0.890	NA	Tissue	qMSP	[39]
*NGFR*; *SEPT9*; *TMEFF2*; bisDNA			0.790	NA	Plasma		
*APC*; *MGMT*; *RASSF2A*; *Wif*-*1*	86.5	92.1	0.927	89.2	Plasma	MSP	[14]
*SFRP2*; *RASSF2*^a^	75.0	89.4	NA	NA	Stool	COBRA, Hi-SA	[40]
*BMP3*; *NDRG4*; *TFPI2*; *Vimentin*	78.0	85.0	0.880	91.2	Stool	QuARTS	[41]
*HLTF*; *HPP1*; *MLH1*	NA	NA	NA	NA	Plasma	qMSP	[42]
*DCC*; *UNC5C*	82.0	NA	NA	NA	Tissue	qMSP	[43]
*HPP1*; *MGMT*; *SFRP2*	93.7	77.1	NA	NA	Stool	MSP	[36]
*IGFBP3*; *miR137*	95.5	90.5	NA	86.0	Tissue	Bisulfite pyrosequencing	[44]
*CNRIP1*; *FBN1*; *INA*; *MAL*; *SNCA*; *SPG20*^b^	94.0	98.0	0.984	95.5	Tissue	qMSP	[45]
*CMTM3*; *MDFI*; *SSTR2*	81.0	91.0	0.920	NA	Tissue	Bisulfite pyrosequencing	[46]
*MGMT*; *RASSF1A*; *SEPT9*	*96.6*	*74.0*	*0.970*	*90.8*	*Tissue*	*qMSP*	*–*

*COBRA* combined bisulfite restriction analysis, *Hi-SA* high-sensitivity assay for bisulfite DNA, *NA* not available for the panel of genes, *QuARTS* quantitative allele-specific real-time target and signal amplification

^a^ Also detects gastric cancer

^b^ Co-methylation of two out of six genes

A potential downside of the use of DNA promoter methylation-based biomarkers for CRC detection is its potential association with a specific molecular subtype, namely MSI-H, which is due to defects in MMR pathway [3]. This system includes genes like *MLH1*, *MSH2*, *MSH6*, and *PMS2* whose expression was screened by immunohistochemistry. Therefore, the loss of expression of any of these genes is considered indicative of defective MMR and, consequently, of MSI-H, whereas the remaining most likely represent MSI-L or MSS tumors. Using this strategy, only 3.7% of cases were disclosed as MSI-H, which is lower than reported in other series, especially those using direct MSI analysis [2, 3]. It is noteworthy, however, that in our series, almost 50% of tumors were localized in the rectum, whereas in most series rectal carcinoma represents less than 30% of CRC. Because rectal carcinoma usually displays a lower frequency of MSI-H cases, as verified in our series, the “over-representation” of tumors localized in the rectum might have decisively influenced the overall frequency. Curiously, CIMP cases were more frequent in the rectum (11.5% vs. 5.6%), with an overall frequency of 8.5%, which is close to the lower end of published series [2, 3]. Owing to the lack of agreement on the best strategy to define a CRC case as CIMP, it is not possible, however, to perform direct comparisons. Nonetheless, it should be emphasized that excepting for *APC*, no significant differences in gene promoter methylation levels were found among MSI-H, CIMP+ or CIN CRC molecular subtypes, nor among different pathological stages or tumor location, in our series. Thus, it may be concluded that the performance of the three-gene promoter panel is likely to be homogeneous across molecular subtypes, and the same applies for primary tumor localization and pathological stage.

We also tested the prognostic value of the five genes promoter methylation status, as this might convey valuable information for clinical decision making. Remarkably, higher *SEPT9* promoter methylation was independently associated with increased DSS in CRC, whereas no prognostic value was depicted for the remainder gene promoters. A meta-analysis that included 14 studies showed that *MGMT* methylation status was not associated with CRC prognosis [47], whereas *RASSF1A* promoter methylation has been associated with poor prognosis, although when assessed in plasma samples [30]. Furthermore, CIMP status also did not disclose prognostic value in our study, which is in accordance with a recent meta-analysis that found no significant effect of CIMP status in DFS or OS in 8 out of 11 and in 13 out of 19 studies previously published, respectively [48]. The same was reported regarding DSS, in 3 of 4 studies considering the classical CIMP panel [8]. Thus, globally, our results are by most of the published literature on the subject.

## Conclusions

Our results indicate that *MGMT/RASSF1A/SEPT9* gene promoter methylation panel accurately identifies CRC, irrespective of molecular subtype and may have a better performance than currently available epigenetic-based biomarkers. Nevertheless, the development of a clinically useful test derived from these results requires assessment of its performance in liquid biopsies, especially blood samples.

## Additional file

**Additional file 1: Table S1.** General features of normal colon and rectum samples used for control purposes. **Table S2.** Primers sequence used for qMSP analysis.

## Abbreviations

AJCC: American Joint Committee on Cancer
CIMP: CpG island methylator phenotype
CIMP-0: CIMP-negative
CIMP-H: CIMP-high
CIMP-L: CIMP-low
CIN: chromosomal instability
CRC: colorectal cancer
CRN: non-malignant colorectal mucosas
FFPE: formalin-fixed paraffin-embedded
MMR: mismatch repair
MSI: microsatellite instability
MSI-H: MSI-high
MSS: microsatellite stable
ROC: receiver operator characteristics
