# Supplementary material for: A novel DNA methylation panel accurately detects colorectal cancer independently of molecular pathway
Source: J Transl Med. 2018 Feb 27;16:45. doi: 10.1186/s12967-018-1415-9 (PMC6389195; doi:10.1186/s12967-018-1415-9)
Supplement: Supplementary file 1 — Additional file 1: Table S1. General features of normal colon and rectum samples used for control purposes. Table S2. Primers sequence used for qMSP analysis. [file 12967_2018_1415_MOESM1_ESM.docx]

**Tables**

| **Table S1**  Basic features of normal colon and rectum samples used. | |
| --- | --- |
| **Characteristic** | **CRN (n = 50)** |
| **Age (years)** |  |
| Mean | 57.50 ± 2.15 |
| Range | 18-85 |
| **Gender** |  |
| Female | 32 (64.0%) |
| Male | 18 (36.0%) |
| **Tissue Location** |  |
| Proximal colon | 19 (38.0%) |
| Distal colon | 16 (32.0%) |
| Rectum | 6 (12.0%) |
| Not Available | 9 (18.0%) |

**Table S2**

Primers sequence used for qMSP analysis.

| ***Gene*** | ***Chrom.***  ***location*** | ***Sequence (5’–3’)*** | ***Size***  ***(bp)*** | ***Annealing Temperature***  ***(ºC)*** |
| --- | --- | --- | --- | --- |
| *ACTB-F*  *ACTB-R* | *7p22.1* | *TGGTGATGGAGGAGGTTTAGTAAGT*  *AACCAATAAAACCTACTCCTCCCTTAA* | *133* | *60* |
| *APC-F*  *APC-R* | *5q22.2* | *TGTGTTTTATTGCGGAGTGC*  *CACATATCGATCACGTACGC* | *139* | *62* |
| *IGF2-F*  *IGF2-R* | *11p15.5* | *CGTCGTTTTTTATTGGTTTC*  *CACACGAATAACCCGCCT* | *153* | *60* |
| *MGMT-F*  *MGMT-R* | *10q26* | *TTTCGACGTTCGTAGGTTTTCGC*  *GCACTCTTCCGAAAACGAAACG* | *81* | *60* |
| *RASSF1A-F*  *RASSF1A-R* | *3p21.3* | *AGCGAAGTACGGGTTTAATC*  *ACACGCTCCAACCGAATA* | *111* | *60* |
| *SEPT9-F*  *SEPT9-R* | *17q25* | *TTAGTTAGCGCGTAGGGTTC*  *ACCTTCGAAATCCGAAATAA* | *140* | *60* |

Abbreviations: bp = base pairs; Chrom. = Chromosome; F = Forward; R = Reverse
